# Lactate-to-albumin ratio and 28 day mortality in hypertensive patients with atrial fibrillation: a retrospective cohort study

**DOI:** 10.1186/s40001-025-03170-6

**Published:** 2025-09-15

**Authors:** Rui Wu, Bo Xing, Zijun Zhou, Yuting Huang, Liming Yu, Huishan Wang

**Affiliations:** 1 School of Life Sciences and Biopharmaceuticals, Shenyang Pharmaceutical University, 103 Wenhua Road, Shenyang, 110016 Liaoning People’s Republic of China; 2 State Key Laboratory of Frigid Zone Cardiovascular Disease, Department of Cardiovascular Surgery, General Hospital of Northern Theater Command, 83 Wenhua Road, Shenyang, 110016 Liaoning People’s Republic of China

**Keywords:** Atrial fibrillation, Hypertensive, Lactate-to-albumin ratio, 28 day, Mortality, Cohort

## Abstract

**Background:**

The lactate-to-albumin ratio (LAR) has emerged as a composite biomarker reflecting metabolic stress and nutritional status. This study aimed to evaluate the association between the LAR and 28 day mortality in hypertensive patients with atrial fibrillation (AF).

**Methods:**

We conducted a retrospective cohort study using the MIMIC-IV v3.1 database. Patients were screened for inclusion based on predefined criteria, resulting in a final cohort of 1087 eligible patients. Mortality within 28 days of ICU admission was the primary endpoint. Statistical analyses included LASSO regression and multivariate Cox regression, receiver operating characteristic (ROC) curve, and Kaplan‒Meier survival curve analyses.

**Results:**

The overall 28 day mortality rate was 22.8% (*n* = 248). Compared with survivors, nonsurvivors presented significantly higher LAR values (0.74 vs. 0.52, *p* < 0.001). Multivariate analyses indicated that the LAR was an independent predictor of 28-day mortality (HR 1.03, 95% CI 1.01–1.06, *p* < 0.05), even after adjusting for multiple clinical confounders. ROC analysis confirmed that the LAR had superior predictive ability (AUC 0.661) compared with other biomarkers. Kaplan‒Meier survival analysis revealed significant differences in mortality between the high- and low-LAR groups (HR 2.55, 95% CI 1.97–3.30, *p* < 0.05).

**Conclusions:**

The LAR is an independent predictor of short-term mortality in hypertensive patients with AF. As a practical and easily applicable biomarker, the LAR holds significant potential for early risk stratification and tailored management in this high-risk population. Our findings underscore the importance of integrating LAR into clinical practice to optimize patient outcomes in critical care settings.

## Introduction

Atrial fibrillation (AF) is the most prevalent cardiac arrhythmia encountered in critically ill patients and is associated with increased morbidity and mortality [1]. Its incidence is particularly high among patients with hypertension, a condition affecting a significant portion of the global population [2, 3]. As chronic hypertension is related to various cardiovascular complications, including AF, the co-occurrence of these conditions complicates clinical management and heightens the risk of adverse outcomes [4–6]. In the intensive care unit (ICU) setting, where patients face acute physiological challenges, understanding the risk factors associated with mortality becomes vital for optimizing care and intervention strategies.

Hypertension is intricately linked with several dysfunctional physiological processes, including left ventricular hypertrophy, endothelial dysfunction, and increased vascular resistance, all of which contribute to the development and exacerbation of AF [7–11]. Hypertensive patients with AF often present with greater clinical severity, further complicating their prognosis [12]. Traditional prognostic indicators such as age, comorbidities, and severity scores have been employed in risk assessment, but they may not fully capture the complexity of the underlying pathophysiology.

In recent years, interest has increased in the use of composite biomarkers that can reflect both metabolic stress and nutritional status, such as the lactate-to-albumin ratio (LAR) [13]. Serum lactate levels are well established as markers of tissue hypoperfusion and metabolic derangement, whereas serum albumin levels serve as indicators of nutritional status and systemic inflammation [14–16]. The combination of these two parameters into a single ratio may provide enhanced prognostic precision, offering insights into the metabolic and nutritional status of hypertensive patients with AF.

Previous studies have indicated that an elevated LAR is associated with poor outcomes in various critical illness scenarios, including sepsis, shock, and heart failure [17–19]. However, the prognostic validity of the LAR in the setting of hypertension and AF has not been thoroughly investigated. Given that both conditions are prevalent in the ICU, their interaction creates a unique pathophysiological environment characterized by increased atrial wall stress, maladaptive remodeling, and heightened metabolic/inflammatory burden, which may affect the prognostic performance of existing biomarkers. Although lactate, albumin, and their ratios have been studied in general ICU populations, no prior research has specifically evaluated the LAR in critically ill hypertensive patients with AF. This targeted focus addresses a clinically important subgroup with distinct risk dynamics, in which accurate early prognostic tools may guide tailored monitoring, resource allocation, and intervention. Identifying patients at high risk for morbidity and mortality is essential for effective clinical decision-making and timely interventions.

This study aimed to evaluate the association between the LAR and 28 day mortality in hypertensive patients with AF. We hypothesize that an elevated LAR will be independently associated with increased short-term mortality and will provide incremental prognostic value beyond traditional clinical and laboratory indicators. By utilizing data from the Medical Information Mart for Intensive Care IV (MIMIC-IV) database, this research aims to clarify the potential of the LAR as a simple yet powerful biomarker for early risk stratification and personalized management.

In summary, understanding the interplay between hypertension, AF, and prognostic biomarkers such as the LAR is crucial for improving patient outcomes in the ICU. This study not only seeks to fill the existing gap in the literature but also aims to contribute to ongoing efforts to enhance risk assessment strategies in critically ill patients facing multiple comorbidities. These findings could have a significant impact on clinical practice, offering pathways toward more tailored and effective care for this vulnerable group. Unlike previous LAR studies conducted in broad ICU populations [20, 21], our work specifically targets critically ill hypertensive patients with AF—a group with distinctive pathophysiological interactions, including combined atrial structural remodeling and vascular stiffness—that may alter biomarker performance.

## Methods

### Data sources

This research utilized anonymized patient data from the MIMIC-IV (version 3.1), a critical care database developed through a collaboration between MIT and Beth Israel Deaconess Medical Center [22]. Covering ICU admissions from 2008 to 2022, the dataset includes a range of clinical information, such as physiological measurements, laboratory results, medication usage, and patient outcomes.

To ensure compliance with ethical standards, all personal identifiers were removed in line with HIPAA deidentification protocols [23]. Patient information was pseudonymized with randomly assigned subject and admission codes, and dates were offset to prevent reidentification.

Before accessing the data, Rui Wu (the first author) completed the Collaborative Institutional Training Initiative (CITI) Program (ID: 69440511).

### Study population

**Fig. 1:**
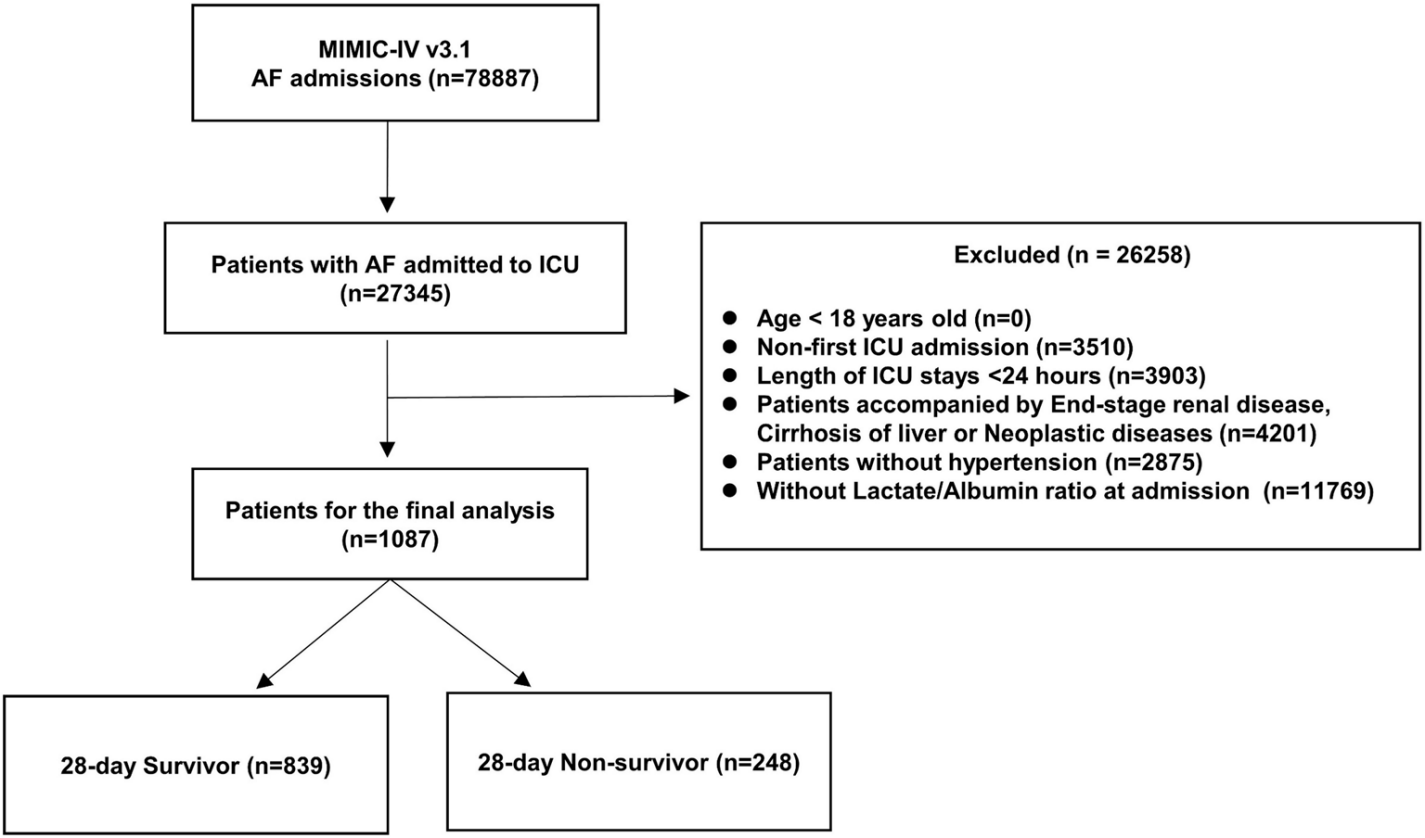
Schematic diagram

Patients diagnosed with AF were identified from the MIMIC-IV database via the International Classification of Diseases codes: ICD-9 code 427.31 and ICD-10 codes I48, I480, I481, I4811, I4819, I482, I4820, I4821, I489, and I4891. These ICD codes encompass paroxysmal, persistent, permanent, and unspecified AF subtypes, ensuring broad capture of AF presentations at ICU admission. A total of 78,887 individuals with AF were initially retrieved, among whom 27,345 had at least one ICU admission. Patients were first identified based on the presence of AF coded at the time of ICU admission. This approach ensured inclusion of individuals with AF during the ICU stay; however, the MIMIC-IV database does not indicate whether AF was the primary admitting diagnosis or a preexisting comorbidity, and our results should be interpreted in this context. Subsequent screening was conducted based on predefined inclusion and exclusion criteria. The following patients were excluded: (1) individuals under 18 years of age at their initial admission; (2) patients with multiple ICU admissions, where only the first admission was retained to avoid duplication bias; (3) patients whose ICU stay lasted < 24 h; (4) those with diagnoses of end-stage renal disease, liver cirrhosis, or malignancies; (5) individuals not classified as having hypertension; and (6) patients lacking serum lactate or albumin measurements within the first 24 h of ICU admission. After applying these criteria, a final cohort of 1087 patients was included for analysis (Fig. 1). The most common reason for exclusion was that serum lactate and/or serum albumin levels were not both obtained within the first 24 h of ICU admission, precluding LAR calculation. In MIMIC-IV, such omissions often occur in patients with milder presentations or different diagnostic work-ups. Consequently, our final analytic sample may overrepresent patients with greater illness severity and more complete laboratory profiling.

The absence of both serum lactate and albumin measurements within the first 24 h of ICU admission was the most frequent reason for exclusion, preventing LAR calculation. We deliberately used this early time window to evaluate LAR as an initial prognostic indicator and to standardize measurement timing, thereby reducing potential confounding from subsequent in-hospital events. In MIMIC-IV, such omissions are more common in patients with milder disease or alternative diagnostic pathways; consequently, our cohort may be biased toward individuals with greater initial illness severity.

### Data extraction

The demographic variables included age, gender, and race. The vital sign data included heart rate, systolic and diastolic blood pressure, mean arterial pressure, and respiratory rate. The laboratory values were also included. The specific comorbidities assessed included cardiovascular disease and chronic diseases. Information on therapeutic interventions and clinical management, including the use of mechanical ventilation, continuous renal replacement therapy (CRRT), endoscopic retrograde cholangiopancreatography (ERCP), and the administration of vasopressors, β-blockers, statins, warfarin, aspirin, and nonvitamin K oral anticoagulants (NOACs), was also captured.

All the data were extracted via structured query language (SQL) queries executed via PostgreSQL (version 17) in combination with Navicat Premium (version 16.3.2) for database management and interface navigation.

### Grouping and endpoint events

Patients were stratified into two cohorts based on 28 day in-hospital survival outcomes: a survivor group (*n* = 839) and a nonsurvivor group (*n* = 248). The primary endpoint of the study was mortality within 28 days following ICU admission.

### Management of missing data

To minimize potential bias arising from incomplete data, variables with more than 15% missing values were excluded from the analysis. For variables with missing rates between 5 and 15%, multiple imputation was employed to estimate and replace the missing entries via the most appropriate imputed datasets [24]. In contrast, when the proportion of missing data was less than 5%, missing values were imputed using either the mean or median.

### Statistical analysis

Continuous variables are presented as the means ± SDs or medians (IQRs), and categorical variables are presented as counts (%). Baseline comparisons utilized independent t tests or one-way ANOVA for continuous variables (based on distribution), whereas Pearson’s chi-square test or Fisher’s exact test was applied to categorical variables.

Prognostic predictors were identified through a two-stage approach. First, univariate Cox regression and LASSO regression were used to select key variables. Next, multivariate Cox regression models were constructed to determine independent risk factors for 28 day mortality. Two adjustment models were implemented: Model 1 controlled for age and gender, whereas Model 2 additionally adjusted for variables including heart rate, respiratory rate, WBC count, LAR, anion gap, BUN, potassium, PT, cerebrovascular disease, CCI, SOFA score, ventilator use, vasopressin administration, beta-blocker therapy, statin use, and warfarin.

The discriminative power of lactate, albumin, the LAR and the SOFA score for mortality prediction was evaluated via receiver operating characteristic (ROC) curves, with the Youden index defining optimal LAR thresholds. Kaplan‒Meier survival curves were used to compare the high- and low-LAR groups, and log-rank tests were used to quantify differences. Restricted cubic splines (RCSs) model potential nonlinear associations between the LAR and mortality risk, supplemented by threshold effect analysis, to detect inflection points. Subgroup analyses validated findings across clinically stratified cohorts.

All analyses were performed in R software (version 4.5.0), with two-tailed *p* values < 0.05 considered statistically significant.

## Results

### Baseline characteristics

A total of 1087 hospitalized hypertensive patients with AF were included in the final cohort, with a 28 day mortality rate of 22.8% (*n* = 248). Significant differences were observed between survivors and nonsurvivors across several clinical domains (Table 1). Compared with survivors, nonsurvivors were older (79.68 ± 10.95 vs. 75.92 ± 11.34 years, *p* < 0.001), and a greater proportion were female (50.4% vs. 42.1%, *p* = 0.020).

**Table 1:** Baseline characteristics

Variables	Total (*n* = 1087)	28 d survival (*n* = 839)	28 d nonsurvival (*n* = 248)	*p*
Admission age, (years)	76.78 ± 11.35	75.92 ± 11.34	79.68 ± 10.95	** < 0.001**
Gender, *n* (%)				**0.020**
Female	478 (43.97)	353 (42.07)	125 (50.40)	
Male	609 (56.03)	486 (57.93)	123 (49.60)	
Race, *n* (%)				**0.018**
White	803 (73.87)	631 (75.21)	172 (69.35)	
Black	98 (9.02)	79 (9.42)	19 (7.66)	
Other	186 (17.11)	129 (15.38)	57 (22.98)	
Heart rate, (beats/min)	86.00 (74.50, 100.00)	85.00 (74.00, 99.00)	91.00 (76.75, 104.00)	**0.002**
SBP, (mmHg)	112.00 (104.00, 123.00)	112.00 (105.00, 123.00)	110.00 (103.00, 121.00)	**0.034**
DBP, (mmHg)	58.00 (53.00, 66.00)	59.00 (53.00, 65.50)	58.00 (52.00, 66.00)	0.464
MBP, (mmHg)	74.00 (68.00, 81.00)	74.00 (69.00, 81.00)	73.00 (66.00, 80.25)	0.083
Resp Rate, (breaths/min)	20.00 (17.00, 23.00)	19.00 (17.00, 22.00)	21.00 (18.00, 24.00)	** < 0.001**
Hematocrit, (%)	29.40 (25.20, 34.05)	29.40 (25.15, 34.15)	29.40 (25.48, 33.30)	0.902
Hemoglobin, (g/dL)	9.82 ± 2.13	9.84 ± 2.10	9.75 ± 2.21	0.557
RDW, (%)	15.00 (14.00, 16.55)	14.90 (14.00, 16.40)	15.50 (14.20, 17.00)	** < 0.001**
Platelets, (× 10^3^/μL)	170.00 (120.00, 238.00)	168.00 (118.00, 233.00)	175.00 (125.00, 252.75)	0.103
WBC, (× 10^3^/μL)	9.60 (6.90, 13.40)	9.10 (6.65, 12.60)	11.25 (7.88, 15.45)	** < 0.001**
Albumin, (g/dL)	3.20 (2.70, 3.60)	3.20 (2.80, 3.70)	3.00 (2.50, 3.40)	** < 0.001**
Lactate, (mmol/L)	1.70 (1.20, 2.60)	1.60 (1.20, 2.40)	2.10 (1.40, 3.60)	** < 0.001**
LAR	0.56 (0.37, 0.90)	0.52 (0.35, 0.79)	0.74 (0.50, 1.27)	** < 0.001**
Aniongap, (mEq/L)	13.00 (11.00, 15.00)	13.00 (11.00, 15.00)	14.00 (12.00, 17.00)	** < 0.001**
BUN, (mg/dL)	28.00 (18.00, 45.00)	25.00 (17.00, 40.00)	35.00 (23.00, 53.25)	** < 0.001**
Calcium, (mg/dL)	7.90 (7.40, 8.40)	8.00 (7.50, 8.40)	7.90 (7.20, 8.30)	**0.022**
Chloride, (mEq/L)	102.00 (98.00, 106.00)	102.00 (98.00, 106.00)	102.00 (97.00, 107.00)	0.360
Creatinine, (mg/dL)	1.20 (0.80, 1.80)	1.10 (0.80, 1.70)	1.40 (0.90, 2.20)	** < 0.001**
Glucose, (mg/dL)	112.00 (93.00, 139.00)	112.00 (94.00, 136.50)	112.00 (89.00, 147.25)	0.769
Sodium, (mEq/L)	137.00 (134.00, 140.00)	137.00 (134.00, 140.00)	137.00 (134.00, 140.00)	0.426
Potassium, (mEq/L)	3.80 (3.40, 4.20)	3.80 (3.40, 4.10)	3.90 (3.40, 4.30)	**0.008**
Abs lymphocytes, (× 10^3^/μL)	0.92 (0.56, 1.42)	0.93 (0.57, 1.39)	0.90 (0.52, 1.45)	0.367
INR	1.30 (1.10, 1.70)	1.30 (1.10, 1.70)	1.40 (1.20, 2.00)	** < 0.001**
PT, (s)	14.50 (12.70, 18.70)	14.30 (12.60, 18.10)	15.80 (13.10, 21.00)	** < 0.001**
ALT, (U/L)	26.00 (16.00, 56.00)	25.00 (16.00, 53.00)	28.00 (15.00, 63.25)	0.367
Bilirubin total, (mg/dL)	0.60 (0.40, 1.00)	0.60 (0.40, 1.00)	0.60 (0.40, 1.00)	0.831
Charlson Comorbidity Index	6.00 (5.00, 8.00)	6.00 (4.00, 8.00)	7.00 (5.00, 8.25)	** < 0.001**
SOFA	6.00 (4.00, 9.00)	6.00 (3.00, 8.00)	8.00 (5.00, 11.00)	** < 0.001**
Myocardial infarct, *n* (%)				0.055
No	809 (74.43)	636 (75.80)	173 (69.76)	
Yes	278 (25.57)	203 (24.20)	75 (30.24)	
Congestive heart failure, *n* (%)				0.278
No	493 (45.35)	388 (46.25)	105 (42.34)	
Yes	594 (54.65)	451 (53.75)	143 (57.66)	
Peripheral vascular disease, *n* (%)				0.787
No	857 (78.84)	663 (79.02)	194 (78.23)	
Yes	230 (21.16)	176 (20.98)	54 (21.77)	
Cerebrovascular disease, *n* (%)				** < 0.001**
No	906 (83.35)	718 (85.58)	188 (75.81)	
Yes	181 (16.65)	121 (14.42)	60 (24.19)	
Chronic pulmonary disease, *n* (%)				0.430
No	687 (63.20)	525 (62.57)	162 (65.32)	
Yes	400 (36.80)	314 (37.43)	86 (34.68)	
Mild liver disease, *n* (%)				** < 0.001**
No	990 (91.08)	779 (92.85)	211 (85.08)	
Yes	97 (8.92)	60 (7.15)	37 (14.92)	
Diabetes, *n* (%)				0.740
No	652 (59.98)	501 (59.71)	151 (60.89)	
Yes	435 (40.02)	338 (40.29)	97 (39.11)	
Renal disease, *n* (%)				0.422
No	707 (65.04)	551 (65.67)	156 (62.90)	
Yes	380 (34.96)	288 (34.33)	92 (37.10)	
Severe liver disease, *n* (%)				**0.012**
No	1082 (99.54)	838 (99.88)	244 (98.39)	
Yes	5 (0.46)	1 (0.12)	4 (1.61)	
Obesity, *n* (%)				**0.036**
No	948 (87.21)	722 (86.05)	226 (91.13)	
Yes	139 (12.79)	117 (13.95)	22 (8.87)	
Ventilation status, *n* (%)				** < 0.001**
No	378 (34.77)	316 (37.66)	62 (25.00)	
Yes	709 (65.23)	523 (62.34)	186 (75.00)	
CRRT, *n* (%)				** < 0.001**
No	1029 (94.66)	806 (96.07)	223 (89.92)	
Yes	58 (5.34)	33 (3.93)	25 (10.08)	
ERCP, *n* (%)				1.000
No	1085 (99.82)	837 (99.76)	248 (100.00)	
Yes	2 (0.18)	2 (0.24)	0 (0.00)	
Vasopressin, *n* (%)				** < 0.001**
No	926 (85.19)	754 (89.87)	172 (69.35)	
Yes	161 (14.81)	85 (10.13)	76 (30.65)	
Beta blocker, *n* (%)				** < 0.001**
No	326 (29.99)	220 (26.22)	106 (42.74)	
Yes	761 (70.01)	619 (73.78)	142 (57.26)	
Statin, *n* (%)				** < 0.001**
No	649 (59.71)	473 (56.38)	176 (70.97)	
Yes	438 (40.29)	366 (43.62)	72 (29.03)	
Warfarin, *n* (%)				** < 0.001**
No	795 (73.14)	586 (69.85)	209 (84.27)	
Yes	292 (26.86)	253 (30.15)	39 (15.73)	
Aspirin, *n* (%)				**0.019**
No	609 (56.03)	454 (54.11)	155 (62.50)	
Yes	478 (43.97)	385 (45.89)	93 (37.50)	
NOAC, *n* (%)				0.121
No	1065 (97.98)	819 (97.62)	246 (99.19)	
Yes	22 (2.02)	20 (2.38)	2 (0.81)	
Length of stay ICU, (days)	4.56 (2.37, 8.41)	5.37 (3.15, 9.42)	3.28 (2.01, 6.54)	< 0.001

*P* < 0.05 are shown in bold

SBP, systolic blood pressure; DBP, diastolic blood pressure; MBP, mean blood pressure; RDW, red cell distribution width; WBC, white blood cell count; LAR, lactate-to-albumin ratio; BUN, blood urea nitrogen; INR, international normalized ratio; PT, prothrombin time; ALT, alanine aminotransferase; SOFA, Sequential Organ Failure Assessment; CRRT, continuous renal replacement therapy; ERCP, endoscopic retrograde cholangiopancreatography; NOAC, nonvitamin K oral anticoagulant

Nonsurvivors presented higher heart rates (91 vs. 85 beats/min, *p* = 0.002) and respiratory rates (21 vs. 19 breaths/min, *p* < 0.001) and lower systolic blood pressures (110 vs. 112 mmHg, *p* = 0.034). Laboratory abnormalities among nonsurvivors included elevated WBC counts (11.25 vs. 9.10 × 10^3^/μL, *p* < 0.001), greater red blood cell distribution widths (15.50% vs. 14.90%, *p* < 0.001), lactate levels (2.10 vs. 1.60 mmol/L, *p* < 0.001), LAR values (0.74 vs. 0.52, *p* < 0.001), and lower albumin levels (3.00 vs. 3.20 g/dL, *p* < 0.001). Other derangements included increased BUN (35 vs. 25 mg/dL, *p* < 0.001), creatinine (1.40 vs. 1.10 mg/dL, *p* < 0.001), anion gap (14 vs. 13 mEq/L, *p* < 0.001), and INR (1.40 vs. 1.30, *p* < 0.001). Nonsurvivors had significantly higher SOFA scores (8 vs. 6, *p* < 0.001) and CCI scores (7 vs. 6, *p* < 0.001). They were also more likely to have cerebrovascular disease (24.2% vs. 14.4%, *p* < 0.001) and mild or severe liver disease (14.9% vs. 7.2%, *p* < 0.001; 1.6% vs. 0.1%, *p* = 0.012, respectively). With respect to treatment, nonsurvivors received mechanical ventilation (75.0% vs. 62.3%, *p* < 0.001), CRRT (10.1% vs. 3.9%, *p* < 0.001), and vasopressin (30.7% vs. 10.1%, *p* < 0.001) more frequently. Conversely, these patients were less likely to receive β-blockers (57.3% vs. 73.8%, *p* < 0.001), statins (29.0% vs. 43.6%, *p* < 0.001), warfarin (15.7% vs. 30.2%, *p* < 0.001), and aspirin (37.5% vs. 45.9%, *p* = 0.019). The median ICU length of stay was significantly longer among survivors than nonsurvivors (5.37 vs. 3.28 days, *p* < 0.001).

### The LAR is an independent risk factor

Univariate Cox regression analysis was conducted for variables that demonstrated statistically significant differences in the baseline characteristics (*p* < 0.05) (Table 2). The analysis revealed a strong association between the unadjusted LAR and 28 day mortality (HR 1.79, 95% CI 1.58–2.03, *p* < 0.001).

**Table 2:** Univariate Cox analysis of risk factors

Variables	*β*	S.E	*Z*	*p*	HR (95%CI)
Admission age	0.03	0.01	4.51	** < 0.001**	1.03 (1.02–1.04)
Gender					
Female					1.00 (Reference)
Male	− 0.31	0.13	− 2.42	**0.015**	0.73 (0.57–0.94)
Race					
White					1.00 (Reference)
Black	− 0.14	0.24	− 0.60	0.550	0.87 (0.54–1.39)
Other	0.40	0.15	2.59	**0.010**	1.49 (1.10–2.01)
Heart rate	0.01	0.00	3.15	**0.002**	1.01 (1.01–1.02)
SBP	− 0.01	0.00	− 1.82	0.070	0.99 (0.98–1.00)
Resp Rate	0.06	0.02	4.25	** < 0.001**	1.07 (1.04–1.10)
RDW	0.08	0.03	3.06	**0.002**	1.08 (1.03–1.14)
WBC	0.06	0.01	6.23	** < 0.001**	1.06 (1.04–1.08)
Albumin	− 0.52	0.10	− 5.24	** < 0.001**	0.60 (0.49–0.72)
Lactate	0.21	0.02	8.52	** < 0.001**	1.23 (1.17–1.29)
LAR	0.58	0.06	9.15	** < 0.001**	1.79 (1.58–2.03)
Aniongap	0.11	0.02	6.25	** < 0.001**	1.11 (1.08–1.15)
BUN	0.01	0.00	5.37	** < 0.001**	1.01 (1.01–1.01)
Calcium	− 0.15	0.06	− 2.42	**0.016**	0.86 (0.76–0.97)
Creatinine	0.15	0.05	2.87	**0.004**	1.17 (1.05–1.30)
Potassium	0.35	0.11	3.23	**0.001**	1.41 (1.15–1.74)
INR	0.17	0.05	3.55	** < 0.001**	1.18 (1.08–1.30)
PT	0.02	0.00	3.72	** < 0.001**	1.02 (1.01–1.03)
Charlson Comorbidity Index	0.14	0.03	5.01	** < 0.001**	1.15 (1.09–1.21)
SOFA	0.12	0.02	7.45	** < 0.001**	1.13 (1.09–1.17)
Cerebrovascular disease					
No					1.00 (Reference)
Yes	0.52	0.15	3.53	** < 0.001**	1.69 (1.26–2.26)
Mild liver disease					
No					1.00 (Reference)
Yes	0.70	0.18	3.90	** < 0.001**	2.01 (1.41–2.84)
Severe liver disease					
No					1.00 (Reference)
Yes	1.68	0.50	3.32	** < 0.001**	5.36 (1.99–14.41)
Obesity					
No					1.00 (Reference)
Yes	− 0.45	0.22	− 2.01	**0.044**	0.64 (0.41–0.99)
Ventilation status					
No					1.00 (Reference)
Yes	0.53	0.15	3.60	** < 0.001**	1.70 (1.27–2.26)
CRRT					
No					1.00 (Reference)
Yes	0.81	0.21	3.84	** < 0.001**	2.25 (1.49–3.40)
Vasopressin					
No					1.00 (Reference)
Yes	1.16	0.14	8.39	** < 0.001**	3.18 (2.43–4.17)
Beta blocker					
No					1.00 (Reference)
Yes	− 0.68	0.13	− 5.31	** < 0.001**	0.51 (0.39–0.65)
Statin					
No					1.00 (Reference)
Yes	− 0.57	0.14	− 4.08	** < 0.001**	0.56 (0.43–0.74)
Warfarin					
No					1.00 (Reference)
Yes	− 0.78	0.17	− 4.48	** < 0.001**	0.46 (0.33–0.64)
Aspirin					
No					1.00 (Reference)
Yes	− 0.32	0.13	− 2.42	**0.015**	0.73 (0.56–0.94)

SBP: systolic blood pressure; RDW: red cell distribution width; WBC: white blood cell count; LAR: lactate-to-albumin ratio; BUN: blood urea nitrogen; INR: international normalized ratio; PT: prothrombin time; SOFA: Sequential Organ Failure Assessment; CRRT: continuous renal replacement therapy

*P* values less than 0.05 are shown in bold

**Fig. 2:**
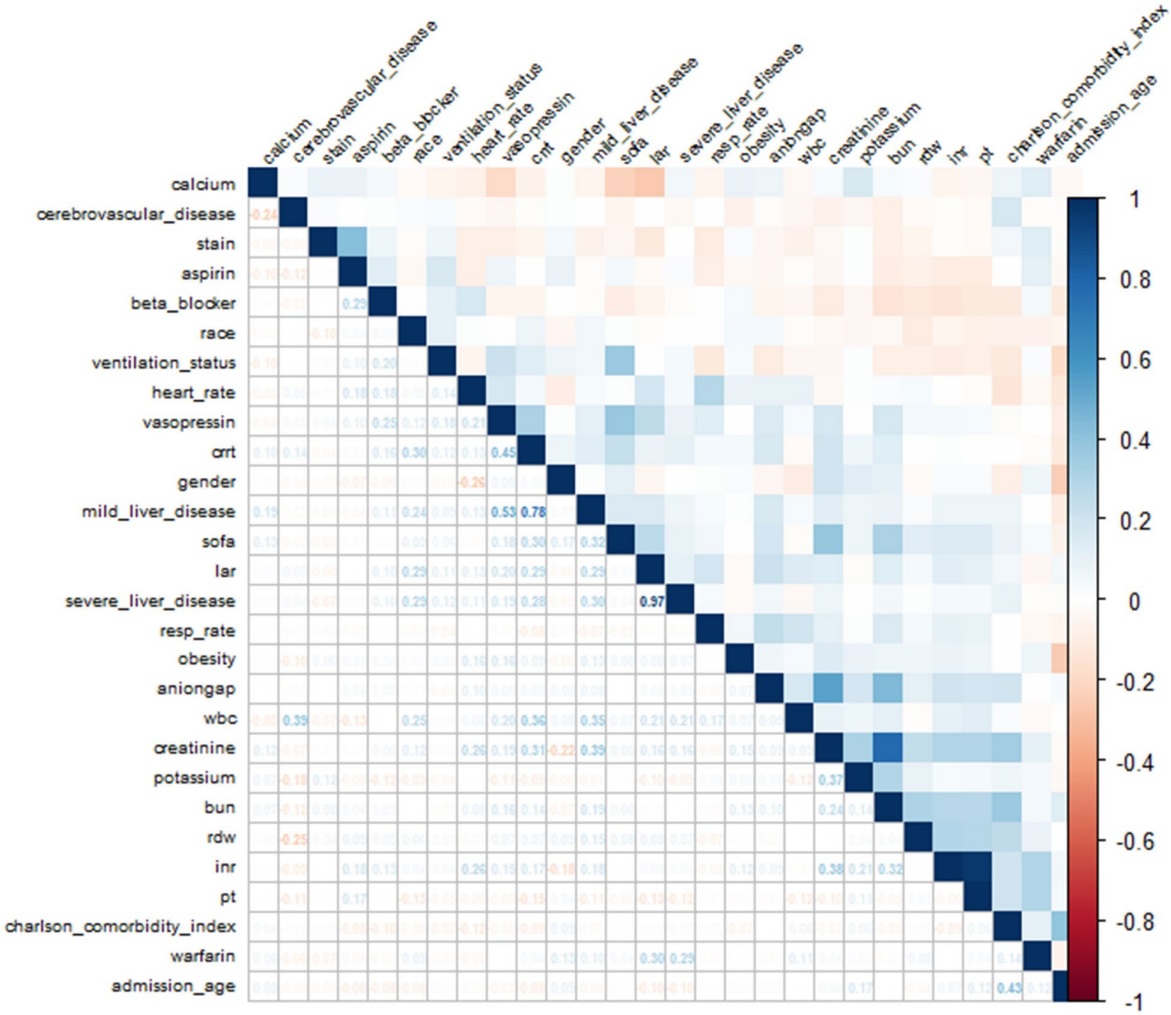
Heatmap of correlations between variables

**Fig. 3:**
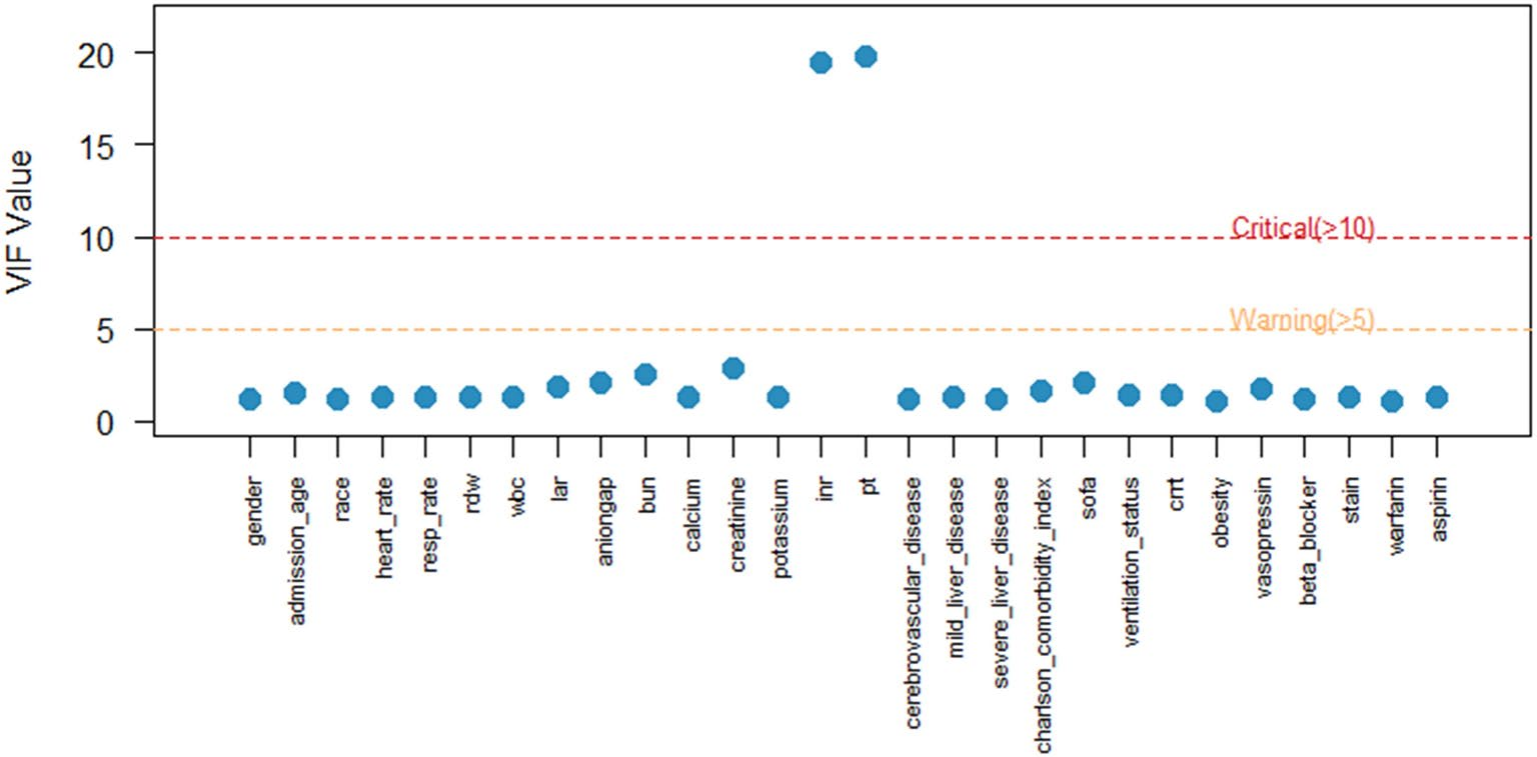
VIF plot for multicollinearity checking. VIF: variance inflation factor

**Fig. 4:**
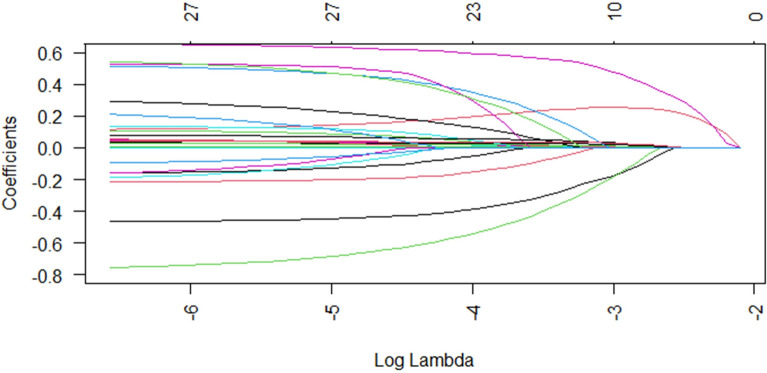
Characterization of the changes in the variable coefficients

**Fig. 5:**
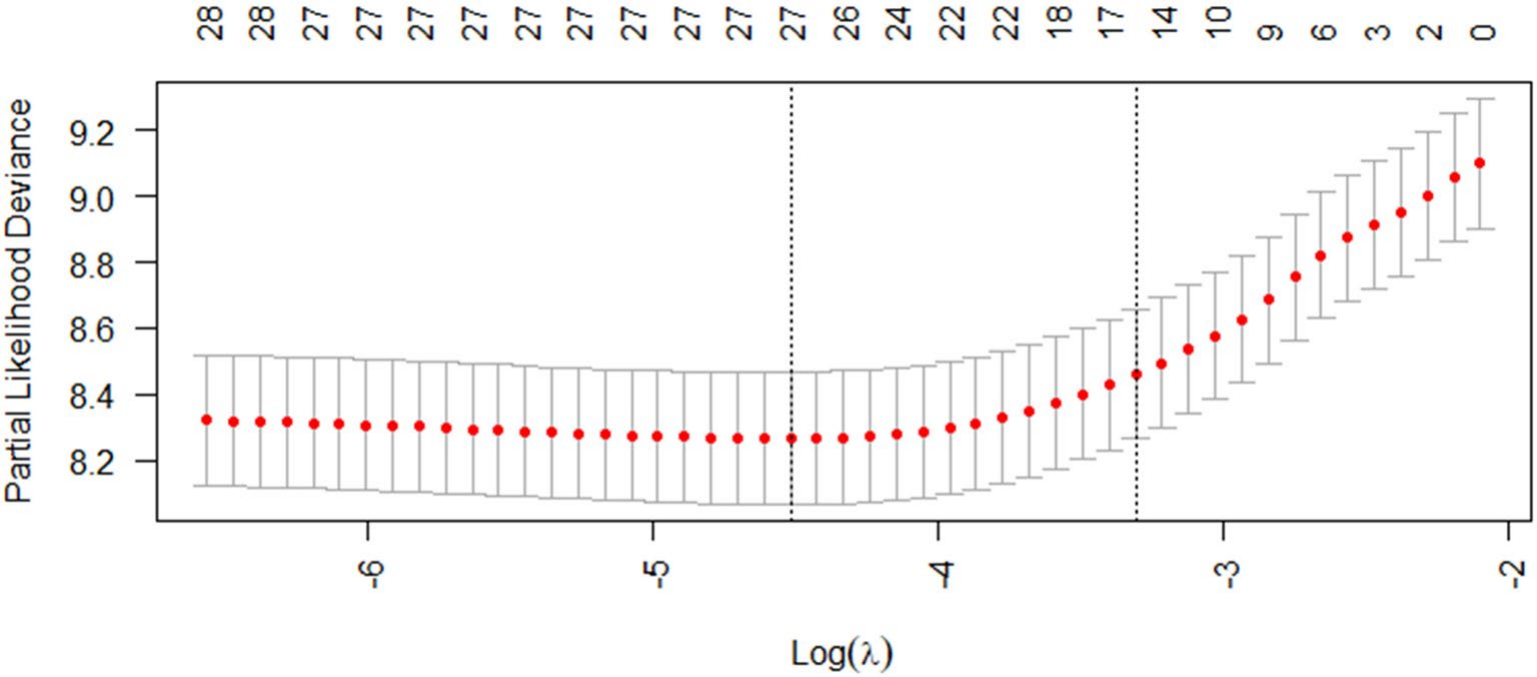
Cross-validation selection process for optimal values of parameter *λ* in LASSO regression models

To mitigate potential overfitting, LASSO regression was applied to variables identified as significant in the univariate Cox models. Prior to LASSO analysis, collinearity was assessed via Pearson correlation matrices and multicollinearity diagnostics (Figs. 2 and 3). Most covariates showed acceptable levels of collinearity, with variance inflation factor (VIF) values less than 5, although a few exceeded this threshold. Following LASSO regression, 17 variables with nonzero coefficients were retained for further analysis (Figs. 4 and 5).

Variables with *p* < 0.05 in both the univariate and LASSO analyses were subsequently included in the multivariate Cox regression model. The results of the multivariable analysis are summarized in Table 3. After adjusting for potential confounders—including age, gender, heart rate, respiratory rate, WBC count, LAR, anion gap, BUN, potassium, PT, cerebrovascular disease, CCI, SOFA score, mechanical ventilation, vasopressin use, and the use of β-blockers, statins, and warfarin—the LAR remained an independent risk factor (Model II: HR 1.03, 95% CI 1.01–1.06, *p* < 0.05).

**Table 3:** Multivariate Cox analysis of risk factors

Variables	*p*	HR (95%CI)	*p*	HR (95%CI)
	Model I		Model II	
Admission age	** < 0.001**	1.03 (1.02–1.05)	** < 0.001**	1.03 (1.02–1.05)
Gender				
Female		1.00 (Reference)		1.00 (Reference)
Male	0.129	0.81 (0.62–1.06)	0.129	0.81 (0.62–1.06)
Heart rate	**0.027**	1.01 (1.01–1.02)	**0.027**	1.01 (1.01–1.02)
Resp rate	0.067	1.03 (1.00–1.07)	0.067	1.03 (1.00–1.07)
WBC	0.075	1.17 (0.98–1.39)	0.075	1.17 (0.98–1.39)
LAR	**0.002**	1.03 (1.01–1.06)	**0.002**	1.03 (1.01–1.06)
Aniongap	0.343	1.02 (0.98–1.07)	0.343	1.02 (0.98–1.07)
BUN	0.472	1.00 (1.00–1.01)	0.472	1.00 (1.00–1.01)
Potassium	**0.019**	1.31 (1.05–1.63)	**0.019**	1.31 (1.05–1.63)
PT	**0.007**	1.01 (1.01–1.02)	**0.007**	1.01 (1.01–1.02)
Charlson Comorbidity Index	**0.020**	1.09 (1.01–1.16)	**0.020**	1.09 (1.01–1.16)
SOFA	0.062	1.04 (1.00–1.09)	0.062	1.04 (1.00–1.09)
Cerebrovascular disease				
No		1.00 (Reference)		1.00 (Reference)
Yes	** < 0.001**	1.74 (1.27–2.37)	** < 0.001**	1.74 (1.27–2.37)
Ventilation status				
No		1.00 (Reference)		1.00 (Reference)
Yes	** < 0.001**	1.85 (1.33–2.58)	** < 0.001**	1.85 (1.33–2.58)
Vasopressin				
No		1.00 (Reference)		1.00 (Reference)
Yes	** < 0.001**	1.93 (1.38–2.71)	** < 0.001**	1.93 (1.38–2.71)
Beta blocker				
No		1.00 (Reference)		1.00 (Reference)
Yes	** < 0.001**	0.58 (0.45–0.76)	** < 0.001**	0.58 (0.45–0.76)
Statin				
No		1.00 (Reference)		1.00 (Reference)
Yes	0.051	0.75 (0.57–1.00)	0.051	0.75 (0.57–1.00)
Warfarin				
No		1.00 (Reference)		1.00 (Reference)
Yes	** < 0.001**	0.46 (0.32–0.65)	** < 0.001**	0.46 (0.32–0.65)

Model I was adjusted for age and gender

Model II was adjusted for Model I plus heart rate, respiratory rate, white blood cell count, lactate-to-albumin ratio, anion gap, blood urea nitrogen, potassium, prothrombin time, cerebrovascular disease, Charlson comorbidity index, Sequential Organ Failure Assessment score, ventilation status, vasopressin, beta blocker, statin, and warfarin

WBC: white blood cell count; LAR: lactate-to-albumin ratio; BUN: blood urea nitrogen; PT: prothrombin time; SOFA: sequential organ failure assessment

*P* values less than 0.05 are shown in bold

**Fig. 6:**
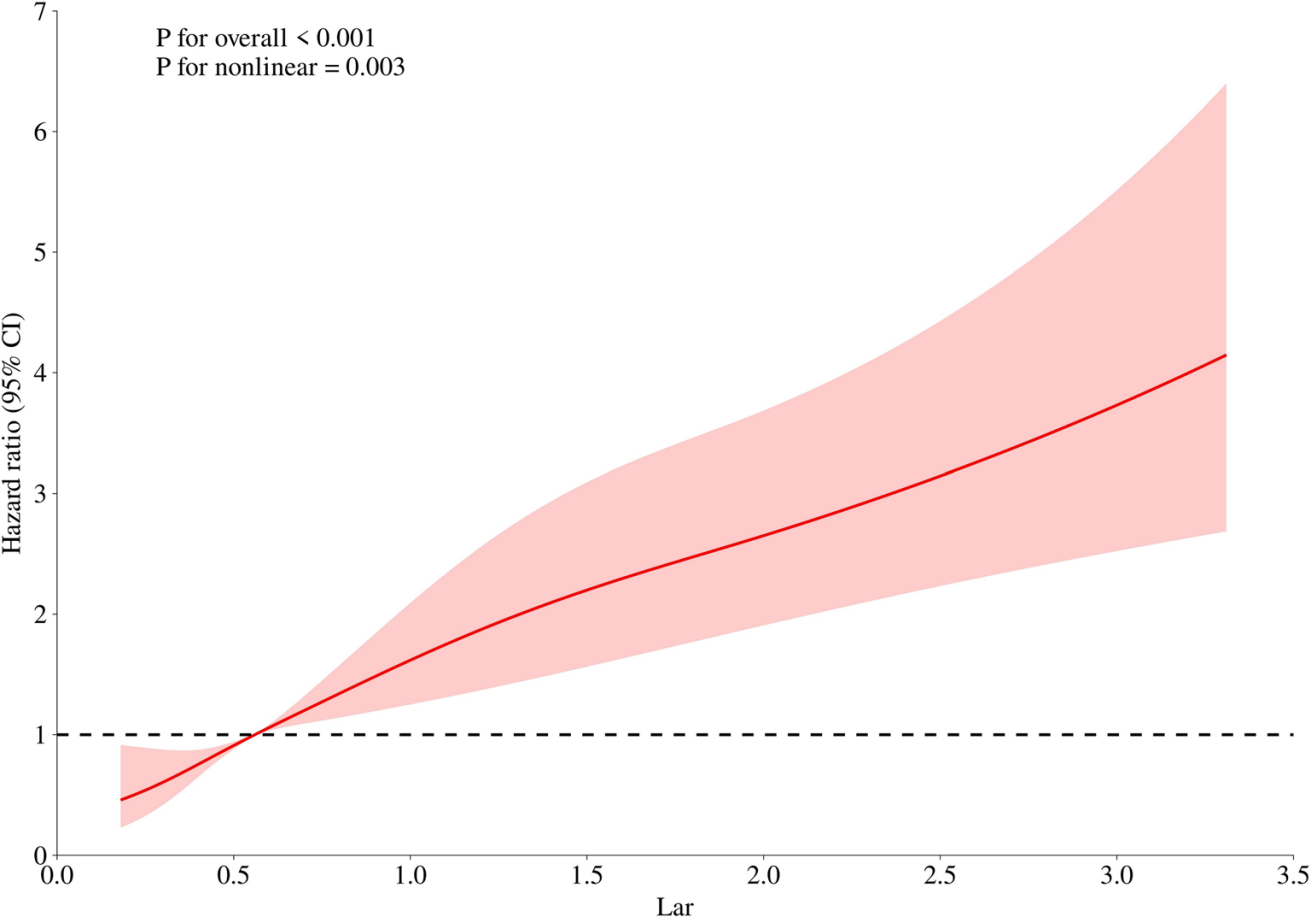
Restricted cubic spline curve analysis for the LAR and mortality. LAR: lactate-to-albumin ratio

To further characterize the association between the LAR and mortality risk, a Cox proportional hazards model incorporating RCS was employed (Fig. 6). This model revealed a significant linear relationship (overall *p* < 0.001) with evidence of nonlinearity (*p* = 0.003). The spline curve demonstrated a dose-dependent increase in hazard, with clinically meaningful risk acceleration observed beyond an LAR threshold of approximately 0.56. These findings underscore the prognostic value of the LAR as a continuous biomarker, where incremental increases above physiological norms (LAR > 0.56) are independently associated with increased mortality risk, without reliance on predefined cutoff values.

### ROC curve analysis and Kaplan–Meier curves

**Fig. 7:**
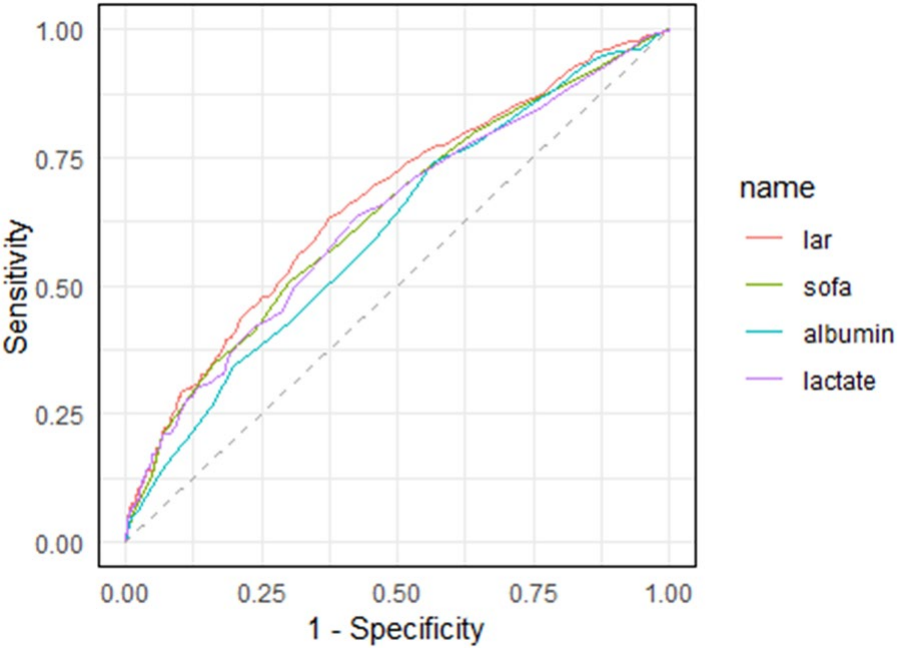
ROC curves. ROC: receiver operating characteristic; LAR: lactate-to-albumin ratio

ROC curve analysis was conducted to assess the discriminatory performance of various biomarkers in predicting 28 day mortality among hypertensive patients with AF (Fig. 7). The LAR demonstrated the highest area under the curve (AUC) (0.661; 95% CI 0.622–0.700) among the evaluated markers, showing only a modest improvement over lactate (AUC 0.630; 95% CI 0.590–0.671), albumin (AUC 0.606; 95% CI 0.567–0.646), and the SOFA score (AUC 0.597; 95% CI 0.597–0.676) (Table 4).At the optimal Youden index cutoff (LAR = 0.605), the sensitivity and specificity were 63.3% and 62.3%, respectively, indicating a balanced performance in both detecting high-risk individuals and excluding low-risk individuals. In comparison, lactate alone (threshold = 1.75 mmol/L) had a slightly lower AUC and similar sensitivity (63.7%) but reduced specificity (57.3%). Albumin, at a cutoff of 3.35 g/dL, demonstrated the highest sensitivity (74.2%) but the lowest specificity (43.4%), whereas the SOFA score (cutoff = 7.5) favored specificity (70.1%) over sensitivity (50.8%). These findings highlight the superior overall discriminatory capacity of the LAR relative to that of individual components or clinical scores.

**Table 4:** The parameters of the ROC curve

Variables	AUC	95%CI	Threshold	Sensitivity	Specificity
LAR	0.661	0.622–0.700	0.605	0.633	0.623
SOFA	0.597	0.597–0.676	7.5	0.508	0.701
Albumin	0.606	0.567–0.646	3.35	0.742	0.434
Lactate	0.630	0.590–0.671	1.75	0.637	0.573

ROC: receiver operating characteristic; AUC area under the curve; LAR: lactate-to-albumin ratio; SOFA: sequential organ failure assessment

**Fig. 8:**
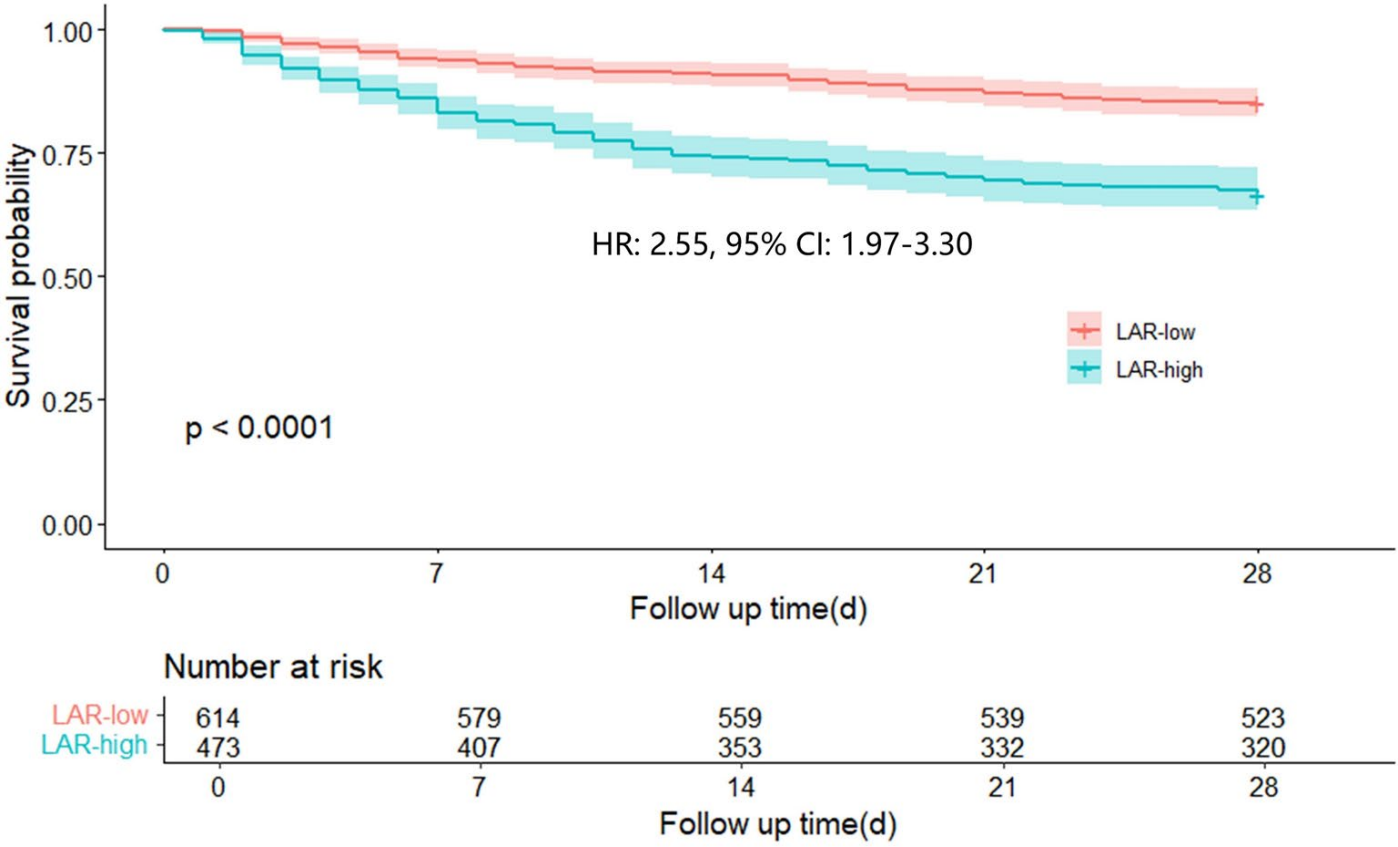
Kaplan‒Meier survival analysis curves. LAR: lactate-to-albumin ratio

The stratification utility of the LAR was Further confirmed by dichotomizing patients at the clinically relevant threshold of 0.605. Kaplan‒Meier survival analysis revealed a significant difference in 28 day mortality between the high- and low-LAR groups (HR 2.55, 95% CI 1.97–3.30) (Fig. 8).

Although the optimal LAR cutoff of 0.605 in our study was derived via the Youden index from ROC curve analysis and is primarily data-driven, it may still offer clinical utility as a preliminary risk stratification tool. Patients with LAR values above this threshold at ICU admission could be considered at higher risk and targeted for closer monitoring, earlier nutritional assessment, and prompt management of metabolic derangements. Nevertheless, we emphasize that this cutoff should be interpreted with caution and ideally validated in prospective, multicenter studies before being implemented as a definitive clinical decision-making criterion. However, in the interim, an LAR ≥ 0.605 at ICU admission may serve as a preliminary alert threshold to identify hypertensive AF patients at higher short-term mortality risk. These patients could be targeted for intensified hemodynamic monitoring, early nutritional assessment, and prompt management of metabolic derangements.

### Subgroup analysis

**Fig. 9:**
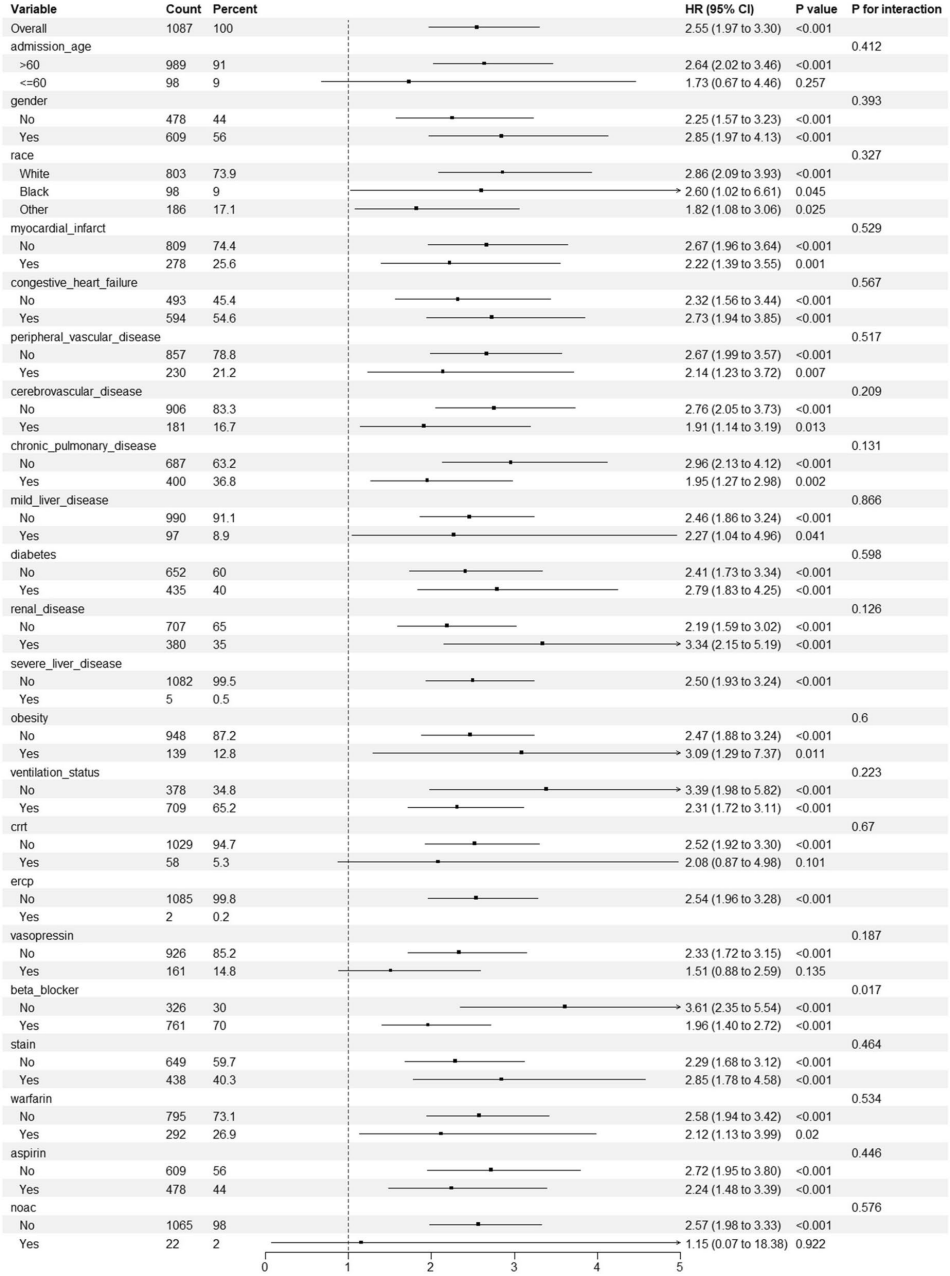
Forest plot of the subgroup analysis for the LAR and mortality. LAR: lactate-to-albumin ratio; CRRT: continuous renal replacement therapy; ERCP: endoscopic retrograde cholangiopancreatography; NOAC: nonvitamin K oral anticoagulant

When the various subgroups were stratified, there was no significant interaction effect between the LAR and the subgroups (Fig. 9).

## Discussion

This study found that the LAR was statistically associated with 28-day mortality in critically ill hypertensive patients with AF. While the predictive strength was modest, these findings highlight the potential value of considering both metabolic derangement and nutritional status when assessing the prognosis of hypertensive patients with AF. This subgroup of patients presents unique challenges: hypertension-associated vascular stiffness and structural cardiac remodeling compound AF-related hemodynamic instability, leading to greater susceptibility to tissue hypoxygenation and systemic inflammation [25, 26]. These characteristics may the prognostic relevance of the LAR compared with more heterogeneous critically ill cohorts.

The clinical implication is that a strong correlation between an elevated LAR and increased mortality highlights how metabolic dysregulation and nutritional deficits can interact to worsen patient outcomes. Elevated lactate levels may indicate tissue hypoperfusion and metabolic acidosis, whereas low albumin levels may serve as a marker of inflammation, malnutrition, and compromised liver function. Notably, even in AF patients without overt metabolic disturbances (i.e., those with normal lactate levels), an elevated LAR can still occur if albumin is reduced, reflecting systemic inflammation or poor nutritional reserves. Conversely, patients with both normal lactate and normal albumin levels have low LAR values, where their prognostic utility might be limited. Future studies stratifying patients by metabolic disturbance status are warranted to better define the predictive role of the LAR across different subgroups. Additionally, this study emphasizes the practicality of using the LAR as a biomarker. In the ICU environment, where rapid assessment and intervention are vital, the LAR provides a straightforward and accessible metric for clinicians. If validated in larger cohorts, it may complement existing scores to enhance risk stratification.

Based on our findings, we propose a preliminary risk-stratification approach using the LAR upon ICU admission. A high LAR (≥ 0.605) indicates compounded metabolic stress and nutritional deficit and should prompt intensified hemodynamic and perfusion monitoring (e.g., lactate clearance tracking, invasive monitoring if indicated), early comprehensive nutritional assessment with the initiation of tailored nutritional support, prompt evaluation and correction of reversible causes of lactatemia, consideration of early multidisciplinary review involving critical care, cardiology, and nutrition teams, and proactive communication with patients and families regarding the prognosis and goals of care. In contrast, a low LAR (< 0.605) warrants continuation of standard ICU monitoring protocols while remaining alert to any dynamic increases in the LAR during the stay. This framework is intended as a decision-support guide rather than a standalone protocol and should be integrated with established severity scores and clinical judgment, with external validation required before formal incorporation into practice guidelines. In practical terms, because lactate and albumin are routinely measured at ICU admission, the LAR can be rapidly calculated and potentially integrated into electronic health records to identify high-risk patients. An elevated LAR could serve as a trigger for multidisciplinary interventions, such as optimizing tissue perfusion, correcting acid–base disturbances, and providing tailored nutritional support. While this concept is appealing, future interventional studies are needed to determine whether actively lowering the LAR translates into improved clinical outcomes.

In addition to the LAR, numerous other biomarkers have been explored for their prognostic value in patients with AF. These include high-sensitivity C-reactive protein (hs-CRP), B-type natriuretic peptide (BNP), and serum creatinine as well as clinical indicators such as age, sex, diabetes, coronary artery disease, and congestive heart failure. Elevated hs-CRP reflects systemic inflammation and has been linked to increased thromboembolic risk and adverse outcomes [27]. BNP indicates ventricular strain and heart failure, and higher levels predict increased readmission and mortality [28]. Impaired renal function, often assessed via serum creatinine, affects anticoagulant metabolism and increases thromboembolic or bleeding risks [29]. Factors such as age and preexisting heart failure further worsen the prognosis [24–26]. While each marker offers valuable information, they generally capture only single dimensions of patient status. By integrating metabolic stress and nutritional status, the LAR may provide a more comprehensive assessment in critically ill patients with AF and hypertension.

Our study on the LAR as an independent predictor of 28-day mortality in critically ill hypertensive patients with AF is consistent with several recent studies exploring various biomarkers in critically ill patients. These studies highlight the growing interest in the use of biomarkers to improve risk stratification and predict mortality in this high-risk population. Huang et al. [30] reported that the blood urea nitrogen-to-serum albumin ratio (BAR) was significantly associated with increased mortality (HR 1.02, 95% CI 1.01–1.03) in patients with AF. Like the LAR, the BAR reflects both metabolic and nutritional derangements. Our study revealed that the LAR had superior predictive power (AUC 0.661) compared with individual biomarkers such as lactate and albumin, highlighting its potential for better mortality prediction in critically ill patients with AF. Xia et al. [31] examined the endothelial activation and stress index (EASIX) and its association with mortality in critically ill patients with AF and reported that higher EASIX levels were correlated with increased short-term and long-term mortality​. EASIX, while a valuable biomarker, requires multiple components, such as lactate dehydrogenase, creatinine, and platelets, making it less practical than the simpler LAR in some clinical settings. This makes the LAR a potentially more accessible and straightforward marker for predicting mortality in critically ill patients with AF.

In another study, Huang and Lin [32] focused specifically on the LAR for predicting in-hospital mortality in ICU patients with AF. They reported that higher LAR values were strongly associated with poor in-hospital survival (HR 2.67, 95% CI 2.39–2.97)​. This finding aligns with our findings, which showed that higher LAR values correlated with significantly increased 28-day mortality. Ma et al. [33] reported that higher triglyceride‒glucose index (TyG) levels significantly increased mortality risk at 7, 15, and 30 days in patients with AF​. Similarly, Zhang et al. [34] confirmed the association between TyG levels and mortality in patients with congestive heart failure and AF​. While these studies emphasize the importance of metabolic disturbances in mortality prediction, the LAR remains distinct by combining metabolic stress (via lactate) and nutritional status (via albumin), offering a comprehensive assessment of the patient’s condition.

In addition to metabolic biomarkers, inflammation-related markers have been explored in critically ill patients with AF. For example, the red cell distribution width-to-albumin ratio (RAR) was studied in patients with chronic obstructive pulmonary disease (COPD) and AF, where a higher RAR was associated with significantly increased 28-day mortality​ [35]. Similarly, the systemic inflammation response index (SIRI) has been linked to poor prognosis in patients with AF, especially those with type 2 diabetes [36]​. Both the RAR and the SIRI reflect the interplay between inflammation and nutritional status, although our study suggests that the LAR may offer a more direct measure of metabolic and nutritional derangements. Several studies have focused on other traditional risk factors for AF, such as age, comorbidities (e.g., diabetes, heart failure), and specific clinical indices. Zhang et al. [34] examined the joint effect of obesity and AF on mortality and revealed that obesity was associated with reduced mortality risk in patients with AF, whereas AF itself increased mortality risk​. Similarly, other studies have highlighted the importance of factors such as the glucose-to-glycated hemoglobin ratio (GAR) in predicting mortality [37]​. However, LAR offers an additional advantage by integrating both metabolic stress (lactate) and nutritional status (albumin), which may provide a more holistic view of the patient's clinical status, especially in critically ill hypertensive patients with AF. Our findings that the LAR is an independent predictor of 28-day mortality are consistent with and build upon previous research examining metabolic and nutritional biomarkers in critically ill patients with AF. Compared with other biomarkers, such as the BAR, EASIX, and TyG, the LAR offers a more straightforward and robust tool for predicting mortality risk, especially in patients with comorbid hypertension.

The strengths of this study include its large sample size, which enhances the reliability and generalizability of our results. The use of the MIMIC-IV database, a comprehensive dataset capturing extensive information across various ICU settings, strengthens our analysis. The statistical methodologies employed, including multiple regression and LASSO analysis, facilitate careful control of confounding variables, providing a clearer picture of the relationship between the LAR and mortality. Our findings have potential practical applications in critical care. Because serum lactate and albumin are routinely measured upon ICU admission, the LAR can be calculated rapidly without additional cost or testing. Early identification of hypertensive AF patients with elevated LAR may prompt clinicians to initiate more intensive hemodynamic monitoring and nutritional optimization, to involve multidisciplinary teams such as cardiology, nutrition, or critical care outreach earlier, and to engage in timely goals-of-care discussions with patients and families at particularly high risk. Although our study is observational, these results suggest that the LAR could serve as a simple, accessible, and effective tool to support timely risk stratification and management decisions, especially in resource-limited ICU environments. However, our study has several limitations. First, as a retrospective analysis, it is inherently subject to selection bias and the constraints of observational data. In particular, only ~ 1.4% of the initial AF cohort met all the inclusion criteria, primarily due to missing lactate and/or albumin measurements within the first 24 h. This low inclusion rate may have preferentially selected for patients with more severe illness, thereby limiting the direct generalizability of our findings to all ICU hypertensive patients with AF. Second, although we included the SOFA score in our multivariable analysis, other widely used critical illness severity indicators such as the APACHE II or SAPS scores were not available in the MIMIC-IV database. The absence of these complementary severity scores may have limited our ability to fully adjust for baseline illness severity across the cohort. Third, while LAR’s AUC was Marginally higher than that of lactate or albumin alone, the differences were modest. We did not conduct NRI, IDI, or decision curve analyses to assess the incremental prognostic value. Consequently, we cannot confirm whether LAR provides a statistically significant advantage over existing markers, and Future studies applying these statistical methods are warranted. Fourth, although all included patients had documented AF on ICU admission, we could not discern whether AF was the primary reason for ICU admission or a concurrent finding. This uncertainty complicates direct extrapolation of our results to situations in which AF is the principal cause of critical illness. Fifth, although our ICD-based strategy included multiple AF subtypes, the database does not permit reliable differentiation between new-onset and chronic AF, nor does it capture complete information on prior rhythm control approaches, catheter ablation history, or antiarrhythmic therapy prior to ICU admission. Similarly, while we reported warfarin and NOAC use, inpatient administration of unfractionated heparin or low-molecular-weight heparin was inconsistently documented in MIMIC-IV, limiting our capacity to assess anticoagulation patterns comprehensively. These omissions May constrain the clinical interpretation of our results. Sixth, we evaluated only LAR values obtained within the first 24 h after ICU admission, with the aim of assessing its utility for early risk stratification and avoiding confounding from in-hospital progression. Other metrics, such as peak, nadir, or discharge LAR, might capture additional prognostic information and therefore warrant investigation in Future research. Furthermore, the exclusion of over 11,000 patients without early lactate and/or albumin measurements could have introduced selection bias, as such tests are more often performed in higher-acuity patients. This missingness pattern may therefore have skewed our cohort toward more severely ill patients, limiting generalizability. Finally, several clinically relevant parameters were unavailable or incompletely captured in the MIMIC-IV, and therefore could not be analyzed. These include echocardiographic measurements (e.g., left ventricular ejection fraction, left atrial size), documentation of AF exacerbations during the ICU stay, and procedural or pharmacologic interventions such as pulmonary vein isolation, cardioversion, and inpatient antiarrhythmic drug use. The lack of these data restricts the clinical depth of our analysis, and future studies should aim to incorporate such parameters for a more comprehensive evaluation.

Even though we controlled for a range of confounding variables, unmeasured factors could still influence outcomes. For example, we did not account for specific treatment regimens, variations in patient management, or different healthcare settings, all of which might impact mortality risk. Moreover, although cerebrovascular disease was included as a comorbidity in our adjusted model, other potentially relevant comorbidities (e.g., diabetes, coronary artery disease, chronic kidney disease) were not retained after LASSO variable selection and were therefore not adjusted for. This may have introduced residual confounding, and future research should consider adjusting for a broader range of comorbidities to further clarify the independent prognostic value of the LAR. Furthermore, while the LAR appears to have substantial prognostic value, it is essential to recognize that it does not replace the need for comprehensive clinical assessment. Each patient’s clinical status should be viewed through a broad lens that incorporates various dimensions of health, beyond just metabolic and nutritional markers.

The promising results from this study signal the need for more extensive and validated research on the LAR as a prognostic marker in various clinical contexts. Moreover, external validation of our findings in independent cohorts and across different ICU settings is essential to confirm their generalizability. Variations in patient demographics, disease profiles, and treatment protocols may influence the prognostic performance of the LAR, underscoring the need for multicenter and international studies. Future studies should focus on validating the predictive power of the LAR across diverse patients to confirm its utility and reliability. Another area of interest is the exploration of how LAR can guide clinical interventions. There is a potential opportunity to examine whether therapeutic strategies aimed at reducing lactate levels or addressing nutritional deficiencies may improve outcomes for hypertensive patients with AF. Longitudinal studies could also be beneficial in understanding how dynamic changes in the LAR are related to clinical outcomes over time. Evaluating whether fluctuations in the LAR correspond with changes in patient status or treatment response could provide insights into its role as a real-time prognostic tool. Moreover, investigating the underlying molecular mechanisms that link lactate and albumin levels to outcomes in patients with AF could offer potential breakthroughs in therapeutic strategies. If we can elucidate how lactate and albumin interact at the cellular level during acute and chronic illness, we might be able to identify targets for drug therapy. Finally, extending research on LAR and similar composite markers to include other high-risk patients—such as those undergoing major surgery, those with acute coronary syndrome, or patients with COPD—could further validate their prognostic capacity.

## Conclusion

In conclusion, our study demonstrated that the LAR serves as a valuable prognostic Marker for 28-day mortality in critically ill hypertensive patients with AF. An elevated LAR is independently associated with an increased risk of short-term mortality, highlighting its utility in risk stratification among this vulnerable population. By capturing the interplay between metabolic stress and nutritional status, the LAR offers a comprehensive assessment that can inform clinical decision-making. Given its predictive power and practical applicability, incorporating LAR into routine clinical practice may enhance early identification of high-risk patients within a uniquely vulnerable subgroup—critically ill hypertensive patients with AF—thereby guiding intensified monitoring, resource allocation, and targeted interventions aimed at both metabolic derangement and nutritional deficits. The simplicity, low cost, and availability of the LAR make it especially attractive for integration into routine ICU admission assessments, ultimately improving management strategies and clinical outcomes in critical care settings. In practical terms, a preliminary cutoff of LAR = 0.605 may help ICU clinicians rapidly flag hypertensive AF patients at the highest early mortality risk, thereby prompting early institution of targeted monitoring, metabolic optimization, and nutritional support. While this threshold requires external validation, integrating the LAR into routine admission assessments could enhance timely, personalized intervention strategies.

## Data Availability

The datasets analyzed during the current study can be obtained from https://physionet.org/content/mimiciv/3.1/.
